# Cutaneous Coccidioidomycosis with Tissue Arthroconidia

**DOI:** 10.4269/ajtmh.18-0700

**Published:** 2019-04

**Authors:** Alexandro Bonifaz, Andrés Tirado-Sánchez, Gloria M. González

**Affiliations:** 1 Department of Dermatology and Mycology, Hospital General de Mexico Dr. Eduardo Liceaga, Mexico City, Mexico;; 2 Department of Microbiology, School of Medicine, Universidad Autónoma de Nuevo León, San Nicolas de los Garza, Mexico

**Figure 1.**
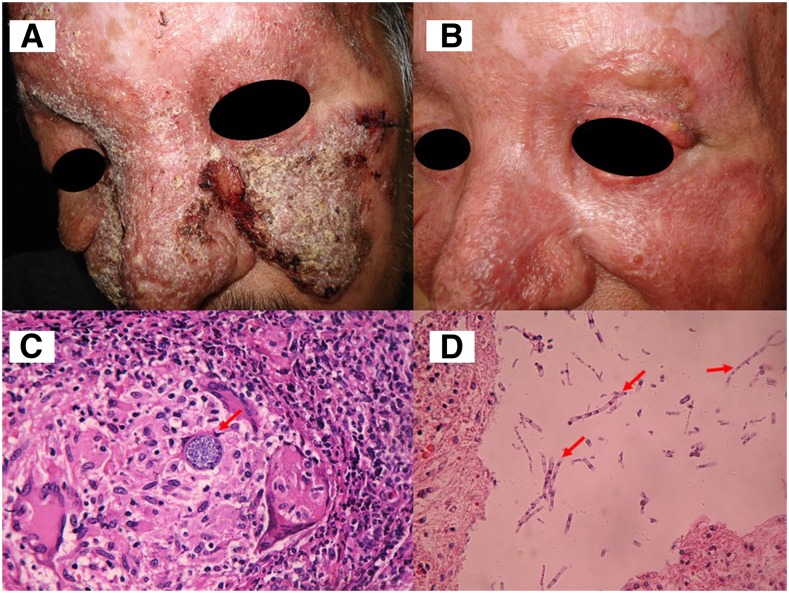
(**A**) Cutaneous coccidioidomycosis (basal). (**B**) Coccidioidomycosis after treatment. (**C**) Histopathology, with granulomatous infiltrate and spherule of *Coccidioides* sp. (hematoxylin and eosin [H&E], 40×). (**D**) Histopathology at the corneal layer with multiple filaments and rexolytic arthroconidia of *Coccidioides* sp. (H&E, 40×). This figure appears in color at www.ajtmh.org.

A 52-year-old man from Tijuana, Mexico, was studied for a 4-year cutaneous disease, characterized by verrucous lesions. He presented with fever and regional adenopathy (cervical, supraclavicular). Chest computed tomography scans showed normal pulmonary activity. He had a history of chronic alcoholism and uncontrolled diabetes mellitus. The presumptive diagnosis was cutaneous tuberculosis. In mycological studies, spherules were observed. Culture and subsequent PCR tests identified *Coccidioides posadasii*. Coccidioidin skin test was positive. Histopathology showed a suppurative granuloma. On histological examination, microabscesses with spherules in the inflammatory infiltrate and hyphae with arthroconidia in the corneal layer belonging to *Coccidioides* sp. were observed (Figure 1). Administration of intravenous amphotericin B and itraconazole achieved clinical and mycological cure.

The largest coccidioidomycosis-endemic area in the world is in the southwestern United States and northwestern Mexico.^1^ Most of the cases are pulmonary^2^ and can present with cutaneous dissemination in ganglionic regions; however, there are primary cutaneous cases (by inoculation) generally with a good prognosis.^3^ *Coccidioides* sp. is a highly infectious dimorphic fungus, which produces spherules with endospores in tissues and hyphae with arthroconidia in the environment or culture media.^4^ The presence of filamentous forms has been previously reported in diabetic patients with pulmonary and neurological diseases,^5–7^ but never in the skin. In this case, the presence of arthroconidia raises the possibility of contagiousness, although person-to-person transmission of coccidioidomycosis has not been previously demonstrated.
